# Best interests and low thresholds: legal and ethical issues relating to needle and syringe services for under 18s in Sweden

**DOI:** 10.1186/s12954-022-00597-6

**Published:** 2022-02-11

**Authors:** Damon Barrett, Frida Petersson, Russell Turner

**Affiliations:** 1 School of Public Health and Community Medicine, Sahlgrenska Academy, University of Gothenburg, Gothenburg, Sweden; 2 Department of Social Work, University of Gothenburg, Gothenburg, Sweden

**Keywords:** Injecting, Youth, Adolescent, Needle and syringe programmes, Child protection, Child rights, Low threshold, Best interests, Law reform

## Abstract

Access for legal minors to needle and syringe programmes raises a number of practical, legal and ethical challenges that traverse clinical practice, child protection and child rights. This article addresses the current legal age restriction on access to needle and syringe programmes (NSPs) in Sweden. Based on legislation and legislative preparatory works, it traces the rationale for retaining an age restriction in the context of a policy priority to improve access for people who inject drugs. Building on threshold theory and child rights literature, the article unpacks the apparent tension between protecting the low threshold nature of service provision, child protection duties of healthcare staff, and the best interests of the child. It explores whether this tension could be alleviated through replacing a legal age restriction for all with best interests assessments for each individual, and discusses the potential ethical and practical challenges involved in such a change.

## Introduction

Approximately 11.3 million people inject drugs globally [47] but injecting drug use among under 18s tends to be a ‘blind spot’ in research, policy and practice [4]. There have been calls for a focus on harm reduction for young people (e.g. [7, 26], but by and large harm reduction services for people who inject drugs have been developed based on, and tend to cater for older participants [14] despite the fact that patterns of use, risks and harms among younger people tend to differ from older counterparts [4, 22]. Work with legal minors raises important ethical concerns that do not arise in the same way with adults, and specific legal issues are brought to bear, including child rights and child protection laws. We use the term ‘legal minors’ to foreground such issues that can be obscured when terms such as ‘young person’ and ‘youth’ are employed and that regularly indicate age ranges crossing a legal, ethical and social threshold into ‘adulthood’. While the term ‘child’ may seem inappropriate for older adolescents, we use it also here for legal purposes. The UN Convention on the Rights of the Child (CRC), for example, applies to everyone under the age of 18 and requires that States Parties take ‘appropriate measures’ to protect children from drug use (Article 33). The UN Committee on the Rights of the Child has consistently recommended ‘youth-friendly harm reduction’ interventions that in turn must be grounded in the principle of the ‘best interests of the child’ [5]. However, the Committee has not elaborated further upon what this recommendation means in terms of law, policy and practice.

Studies focusing on the legal and policy environment for harm reduction work with this age group are rare [20]. See, however, [15, 48]. A technical brief has, however, been produced by the World Health Organization on injecting drug use among young people aged up to 25, which includes issues specific to under 18s [49]. Some countries have dedicated policy guidance for minors’ access to NSP. Some adopt age restrictions in legislation [15], which the WHO recommends that states ‘consider’ removing ([49], p. 19). According to the International Guidelines on Human Rights and Drug Policy [50], States should ‘Remove age restrictions on access to harm reduction services where they exist, and instead ensure that in every instance in which a young person seeks access to services, access is determined based on the best interests and evolving capacity of the individual in question’ (Guideline II.1.1.iii). This is perhaps easier said than done, as is the case with many guidance documents and human (and child) rights standards. It requires attention to why such laws are in place and the details of what might replace them.

This article is part of a larger multi-country comparative project focusing on injecting drug use among legal minors through the lens of the best interests of the child. Part of the project involves legal and policy mapping across the three focus countries, which in turn will inform later qualitative research with young people who inject drugs, and service providers.^1^ The article focuses on the legal age restriction of 18 on access to needle and syringe programmes (NSP) in Sweden, which emerged from the mapping as an area of practical and ethical tension.

Among scholars, practitioners and advocates, Sweden’s past ambivalence, an even antagonism, towards harm reduction, is well known. The official policy for many decades has been the pursuit of a drug-free society, with the protection of young people being central to this goal, and within which harm reduction is not an easy ideological fit [13]. However, in the past decade, especially in response to hepatitis C and overdose deaths, harm reduction interventions in Sweden have nonetheless moved forward. Naloxone distribution has begun during this time, and from only two official NSP in just one region back in 2006, today there is at least one NSP in almost all twenty-one regions of the country [44].

While the numbers of legal minors who inject drugs at any one time will likely be very low in Sweden, any exclusion from healthcare based on age requires a solid rationale. This article looks to the official rationale for the age restriction on NSP from the preparatory works of the relevant legislation. From these sources, we see that the age restriction in Sweden was not due to an ambivalence about harm reduction, or a worry about the appropriateness of the service for minors. It was officially justified by a need to protect the ‘low threshold’ nature of NSP, when all child protection duties were also taken into account. The article begins by briefly introducing the situation regarding injecting drug use and related health harms in Sweden. It then turns to the relevant legal framework for child protection, child rights, healthcare and NSP. It goes on to trace the rationale for retaining the age restriction on access to NSP, and to critically assess this rationale through the lens of ‘the best interests of the child’ [12] and threshold theory [11]. It then explores whether a recognition of lower thresholds for under 18s and best interests assessments can provide more flexibility for access, while remaining true to child protection obligations. Ethical and practical challenges relating to indeterminacy, ascertaining competence, and staff perspectives and capacity are discussed.

## Methods and materials

The material for this article has emerged from a wider legal and policy mapping exercise relating to injecting drug use among legal minors in Sweden, Switzerland and Wales. The mapping contributes to investigating one of our research questions: ‘In what ways may the legal and policy environments be conducive to, or act as barriers to realising the needs and rights of young injectors?’ Initial workshops were held to discuss key legal and policy issues that would be investigated in each country, leading to a list of questions across five thematic domains: overview of the legal system; child law and best interests; organisation of health and social care; drug laws and the criminal code; drug treatment (voluntary and involuntary); and harm reduction. The mapping began with exploratory work in secondary sources, such as leading textbooks and relevant government reports to begin investigating the relevant thematic domains. Initial legal and policy sources relevant to each question were collated in Excel, organised by theme. Further sources were identified as they were referred to within the initial list (e.g. references to amending legislation). A further workshop refined the questions into legal/policy ‘constructs’ that could be documented and ultimately compared across each jurisdiction (see [2]). Taking the initial list of legal and policy documents and the various constructs as the starting point, more in-depth analysis was conducted.

In Sweden, the relevant ‘propositions’ for each piece of primary legislation were also downloaded. These are referred to below as (Prop. + number/year) and are official government reports that document the preparatory work for the legislation, including the official proposal for legislation by the government, the rationale for such proposals, and input from various actors. They are an important source of legal interpretation in the Swedish legal system ([8], pp. 45–48). These primary sources were read in depth, and relevant passages relating to injecting drug use and age were extracted.

Searches were also conducted on the Government’s website where official documents are publicly available. Search terms included ‘sprutbyte’ OR ‘sprututbyte’ (both used for NSP) AND ‘åldersgräns’ (age limit) OR ‘ålder’ (age). This produced new results, in the form of certain parliamentary motions, but did not add significantly to the above, more iterative and structured process. The websites of the relevant government authorities were also searched, and key informants within the agencies were contacted for advice on key policy documents or existing guidelines to include. Again, relevant passages from these documents were extracted.

A legal/policy memo was produced based on the mapping, which described the legal and policy situation in Sweden, including age restrictions on access to NSP and related law and policy. That, in turn, exposed legal, ethical and practical tensions and inconsistencies requiring further critical exploration. This article therefore takes the legal mapping as an entry point for a critique of the current situation grounded in the primary legislation, the preparatory works, policy documents, and child rights principles that frame our wider research project.

## Injecting drug use, health harms and access to healthcare for minors

There is no reliable population size estimate for people who inject drugs in Sweden, though in 2011 it was estimated to be approximately 8000 [16], p. 10). Age and circumstances of initiation are also unclear, as they are in most countries (see [4]). According to the Public Health Agency of Sweden, the average age of initiation into injecting is around 18–19 ([16], p. 15). But interviews with over 150 young people in compulsory institutional care found 20 had experience of injecting drug use, with an average age of initiation at 16. More girls than boys reported injecting ([36], p. 30). This followed earlier research which had shown that 18% of under 18s in compulsory care reported injecting drugs in the past year, with greater risk behaviours, and lower health knowledge than older counterparts [37]. Hepatitis C (HCV) prevalence is high in people who inject drugs in Sweden, and it has been estimated that within two years of initiation of injecting, a person will likely have contracted the virus [16], p. 16). A quarter of all reported HCV cases in 2014 were young people aged 15–24, 86% of which contracted the virus through unsterile injecting equipment. As the Public Health Agency testified at a hearing on the appropriate age for access to NSP, ‘this gives an indicator of ongoing recruitment of young people into injecting drug use, and ongoing infection of hepatitis C among the youngest’ ([41], p. 16).

Beyond sterile equipment, NSP in Sweden offer HCV testing and treatment referral. While hepatitis is one important health risk that can arise from injecting drug use, there are many other health harms, including other blood-borne viruses, the development of wound abscesses and overdose risk. A wider array of services is therefore offered via NSP. Indeed, it is important to emphasise that NSP is not only interventions for preventing the spread of blood-borne viruses and providing primary healthcare. They can function as important aspects of comprehensive health and social care for marginalised people, as the Swedish Government has long recognised ([33], pp. 124–125, 135). However, by law those under the age of 18 who inject drugs are not permitted to exchange injecting equipment ([23], 6§). Hence, minors who inject—and who may have greater health needs—potentially have more limited access to healthcare. Moreover, in being excluded from the NSP an important point of contact for social care may be lost.

## Legal framework

### Minority status

According to the Family Code, anyone under the age of 18 in Sweden is a ‘minor’ ([17], chapter 9, 1§). Other laws, such as the Social Services Act, confirm this by defining a child as anyone under the age of 18 ([40], chapter 6, 2§). Those over the age of 15 are, however, in various situations presumed to have the capacity to consent. For example, the age of consent for sex is 15 ([10], chapter 6, 4§), as is the minimum age of criminal responsibility (ibid chapter 1, 6§). Consent is also required for random school drug testing [21]. As a general rule, healthcare cannot be provided without consent, except for specific situations (e.g. unconsciousness) ([31], chapter 4). Children’s views about their own healthcare must be sought, depending on their age and maturity (Ibid, chapter 4, 3§). In principle, as the child matures, their views on matters affecting them should be given increasing weight.

### Needle and syringe programmes, and needle purchase

NSP is run by regional authorities as part of their healthcare responsibilities. While there is a general Healthcare Act (Hälso- och sjukvårdslag 2017:30), there is also a dedicated Act on the exchange of needles and syringes, adopted in 2006 [23]. NSP is primarily seen as being for the control of infectious diseases (ibid, 1§), but it is accepted that this requires a more holistic approach than merely needle exchange ([36], p. 23). There are various conditions of access and legislative requirements, including a general requirement of one-to-one exchange of equipment, but the most relevant for this article is that those under the age of 18 are not permitted to exchange needles ([23], 6§). Due to the dedicated NSP Act, the actual exchange of needles is not legally defined as ‘healthcare’ for the purposes of the Healthcare Act, unlike other services that the NSP may provide, such as hepatitis testing ([36], p. 27). Thus, needle exchange is regulated by the NSP Act separate from other forms of healthcare provided at the same sites. What this means is that, strictly speaking, someone under 18 can attend the service and receive these other forms of healthcare, but cannot exchange needles.

Needles may be sold in pharmacies in Sweden, but if the person is under 20, this may only be for the person’s own or a family member’s documented health condition ([24], 4§). A breach of this provision may result in a fine for the pharmacy involved (ibid 9§). The age restriction of 20 for sales was seen as appropriate against the background of the same lower age limit being applied at the time to needle exchange ([34], p. 23). However, as this has now been reduced to 18 (discussed below), the two pieces of legislation are out of sync ([36], p. 36).

### Child rights and the best interests of the child

Issues relating to children are addressed across the Family Code, the Social Services Act, the Healthcare Act and other issue-specific laws/regulations. The CRC has been brought into domestic law in its entirety and entered into force in 2020 [25]. Thus, the child’s right to health (Article 24) and right to protection from drugs (Article 33) are now recognised in legislation. The child’s right to be heard (including in healthcare) is protected via Article 12 of the CRC as well as Sweden’s Patient Act ([31], chapter 4, 3§).

‘The best interests of the child’ is a long-standing legal concept. As Article 3.1 of the CRC states: ‘In all actions concerning children, whether undertaken by public or private social welfare institutions, courts of law, administrative authorities or legislative bodies, the best interests of the child shall be a primary consideration.’ The principle has long been a feature of Swedish law, and since the ratification of the CRC in 1990 has been explicitly incorporated via ‘portal’ provisions into various laws and regulations ([39], p. 986. See also [38]). These establish the best interests of the child as a guiding principle in the relevant area covered by the legislation, and tend to state that the best interests of the child shall be given ‘special consideration’ (e.g. [51], chapter 5, 6§; [31], chapter 1, 8§). According to the Healthcare Act, the principle must be given special consideration ‘when’ healthcare is given to children. As outlined above, the provision of sterile-injecting equipment has its own legal framework in the NSP Act, which omits the best interests principle given the use of an age restriction.

### Child protection: the duty to report regarding children at risk

One more legal consideration is central to the topic at hand. According to the Social Services Act, authorities and staff working with children in healthcare have a duty to report to the Social Welfare Board if they have knowledge or suspect a child is being harmed or is at risk of being harmed ([40], chapter 14, 1§; see also [43]. Reponses to such reports may involve placing a child in compulsory residential care. In such cases, the best interests of the child are not just to be given ‘special consideration’, they are ‘decisive’, reflecting the gravity of the decision for individual children ([40], chapter 14, 1a§, Care of Young Persons (Special Provisions) Act [9] 1§). NSP staff are subject to this legal requirement, and it is generally accepted that injecting drug use among someone under the age of 18 would trigger the duty to report ([36], p. 32).

## Tracing the rationale for the age restriction on NSP

Focusing on legislative debates regarding age of access, we begin with the initial NSP Act, adopted in 2006. However, it is important to note that idea of NSP had long been controversial, and debates had been ongoing for many years leading up to the Act. These related more to clashes in values (e.g. class, nationalism, individualism) and how the problem of drugs was therefore framed (e.g. social justice, disease) than empirical evidence [13]. The stated aim of the proposal for an NSP Act was to improve access to NSP beyond the two existing services that had run since the late 1980s in Lund and Malmö on an experimental basis. It aimed to improve services for ‘heavy drug users’ and to address injecting-related health harms ([33], pp. 123–128, 131–132). The original proposal was for an age restriction of 20 years, based on two main reasons: need and appropriateness. First, the age of those currently accessing the two existing services was considerably higher, with an average age of 40 among men and 37 among women. The average age had, moreover, been gradually increasing over the years, and the lowest age of anyone accessing the two services was 20. Injecting drug use among under 20s was seen to be very unusual ([33], p. 135); thus, there was no perceived need. Second, the Government’s view was that society had a duty to not allow teenagers to begin injecting drugs in the first place. The most appropriate intervention was through social services, and compulsory institutional care if needed (ibid p. 136). In other words, NSP was inappropriate. This is in keeping with a scepticism among opponents at the time about the appropriateness of NSP for any age group, when other interventions were available [13].

While a number of NGOs and the Medicines Authority were of the view that need and not age should determine access [33] p. 134), the age restriction of 20 was ultimately agreed among the various regional authorities, municipalities and others taking part in the debates, including by the existing two NSPs. However, it was accepted that things may change in future, and the door was left open for amendment if future evaluations deemed it necessary (ibid p. 136).

The NSP Act was, indeed, revised ten years later. In Sweden’s legal framework, regional authorities have responsibility for healthcare, and municipalities have responsibility for social services, education and other issues. Though NSP is therefore run by regional health authorities, the original NSP Act required the support of the municipality in which a service would be opened, granting them an effective veto. For a decade, this prevented the initiation of an NSP in Gothenburg, Sweden’s second largest city, due to a long-standing antagonism to NSP within the municipality. This legal hurdle is important to note because it was a main impetus for the review process, aiming to improve access to NSP nationwide [45]. Among the reforms on the table was the reduction of the age restriction then in place from 20 to 18. Inevitably, the question arose as to whether an age restriction was appropriate at all.

The rationale for reducing the age during the 2016 review was on the evidence that there were those under the age of 20, and indeed under 18, injecting drugs and being at risk of infection with blood borne viruses (the main rationale for the law, and recognised in parliament – see [27]). Reducing the age of access would also increase opportunities to motivate younger people into treatment [28]. In contrast to the reasoning ten years before (lack of need), the Government now stated that ‘…it is not crucial for the proposal to lower the age limit if the group is large or small, and just as with all types of drug abuse, it can be assumed that there are data gaps’ ([36], p. 30, 31). More important now, and in keeping with a policy focus on improving health equality, was that ‘[Every individual who can be protected against infectious diseases and who can thus be prevented from spreading these diseases is important. Young people are particularly important in this regard’ (ibid, p. 31; see also [28]). Indeed, some young people under the age of 20 had already contacted NSP services. All NSP providers that fed into the hearings now saw the age restriction of 20 as a ‘limit’ on their official ‘disease prevention mandate’ ([36], p. 29).

There was disagreement, however, as to whether to lower the age limit to 18 ([41], p. 34 & 35). Various universities, NGOs, municipalities and regions agreed with lowering the age. But some important objections and counter-points are worth noting. Gothenburg City Council disagreed on the basis of the State’s obligation to protect children from drugs under the CRC. According to the Council, it was ‘important to clarify that a child perspective should guide needle exchange based on Article 33 of the Convention on the Rights of the Child’, and therefore, ‘the age restriction on needle exchange should not be reduced to 18 years ([36], p. 32)’.It is unclear why this argument was relevant, however, as the CRC does not apply above the age of 18. Nonetheless, the key point in calling for a ‘child perspective’ recalls concerns about the appropriateness of the service for this age group.

The City Council in Örebro (Sweden’s sixth largest city) also disagreed with lowering the age to 18. In its view, it was more important to provide treatment than to lower the age. Those aged 18–20 should in the first instance instead be offered opportunities to stop injecting ([36], p. 32). Other municipalities, regions and NGOs felt that the Law on Care for Young Persons was the most appropriate route, including compulsory institutional care. The representative body for Sweden’s regions and municipalities felt that reducing the age on access to NSP would be ‘inconsistent with society’s duties’ under the Law on Care for Young Persons ([41], p. 34 & 35). Social Services, while not objecting to lowering the age restriction as such, stated in a hearing that young people who inject drugs were ‘already known’ to them ([36], p. 31).The Government recognised this, agreeing that these young people fell mainly under the mandate of Social Services. Legal minors who inject would be dealt with via the Care of Young Persons (Special Provisions) Act [9], which results in an assessment for being taken into care by Social Services. But Social Services do not provide NSP, which are under the authority of the regional health bodies. Implied within the argument is that other services, including being taken into care, would obviate the need for NSP access. Here we see the same concerns that had arisen earlier about the appropriateness of NSP versus other interventions, but focused solely on young people. The review, after all, was aiming to increase access to the service, which was clearly by now accepted as appropriate in general.

On the other side of this debate, Lund University argued for no age restriction at all [41], p. 34), as did Stockholm NSP ([36], p. 32). This mirrored earlier interventions in 2006 to the effect that need, and not age, should determine access. They were supported by some NGOs, but were outnumbered by the remaining NSP providers and other stakeholders that preferred an age restriction of 18.

The age was ultimately reduced from 20 to 18, with the government recognising that there was injecting drug use among those under 20, and a public health and primary care objective in increasing NSP access. But while the above objections all fed into the hearings, the retention of the age restriction at 18 was not due to concerns about the appropriateness of under 18s receiving sterile needles (implied by the city of Gothenburg), or that other services should be a priority (stated by others). It was to protect the ‘low threshold’ nature of the NSPs themselves. This had not arisen in 2006. Central to this decision was the duty to report under the Social Services Act. NSP staff were ‘assumed to have knowledge of if and when they need to act upon this duty’ which would ‘normally’ be required if encountering under 18s who inject ([36], p. 32). The view was taken that this would bring an element of coercion into what should be a low threshold service. According to the Government: ‘[A]s it is important that low-threshold activities work on the basis of voluntariness and dialogue rather than coercion and reporting… the Government considers that it would be inappropriate to allow the activities to include persons where notification is a mandatory requirement’ (ibid, p. 32). Stockholm NSP disagreed, stating that the key issue was trust ([36], p. 32). If the duty to report would breach confidentiality and bring coercion into the programme, then a core tenet of the model would be damaged. However, the solution to the concern about trust is transparency and dialogue. The duty to report situations of children at risk under the Social Services Act, they argued, should not be an impediment to taking in under 18s, so long as there was informed consent ([36], p. 32).

Thus, what we see in relation to NSP is that a general age-determined standard for access for all took precedence over subjective decisions by staff in dialogue with individual clients. This can be explained in terms of defending the low threshold nature of the services as a value, but it is one that could conflict with the best interests of the individuals subject to the age restriction.

## Defining low thresholds

While the term has long been employed to refer to drugs services, especially harm reduction, approaches to ‘low thresholds’ differ. For some, it is about not requiring abstinence as a precondition of access, or not primarily focusing on treatment. For others, it is about reducing barriers for access (of which such a treatment focus could be one) including bureaucratic or administrative barriers. Building on earlier work, Edland-Gryt and Skatvedt [11] identify four types of threshold that must generally be crossed for access to services. The *registration threshold* refers to the fact that ‘all offers of help and assistance in society at large are based on the clients’ initiative and their willingness to register themselves as a person in need of help.’ The *competence threshold* refers to clients’ ability to ‘put forward their needs and requests in a way that staff understand and can act upon’. The *efficiency threshold*, meanwhile, relates to clients who are unable to access services, or are rejected, due to service providers views, emotions or preferences for how the services should be managed ([11], p. 258). And finally, there is the *trust threshold*, which Edland-Gryt and Skatvedt saw as necessary for crossing the previous thresholds, but was itself perhaps the most difficult to cross. It is therefore worth recalling the emphasis placed on trust by the Stockholm NSP in arguing against the age restriction.

Based on a review of the literature, and building on the four thresholds, Mofizul Islam and colleagues have set out three essential criteria for a drugs service to be seen as low threshold. First, drug users should be *the* or at least *a *primary target group. Second, abstinence should not be necessary. And third, barriers to access should be reduced as far as possible. For example, through ‘outreach programs, an inviting atmosphere and effective client engagement, anonymous and confidential service delivery, assertive referral with support for referral uptake, free-of-cost and/or tailored services, peer support, integrated service modalities and support to help reduce personal barriers to healthcare access’ ([30], p. 221). With these criteria in mind ‘[N]ot all harm reduction services are low-threshold, whereas most low-threshold services also have a harm reduction orientation’ (ibid).

With these criteria in mind, were Swedish NSP low threshold to begin with, to the extent that required excluding under 18s? Clearly people who inject drugs are the primary focus, and although NSP must by law work to motivate clients to enter treatment ([23], 1§), abstinence from drugs is for obvious reasons not a condition of access. While there are no scientific studies to date, internal surveys from regional health authorities indicate high levels of satisfaction with NSP.^2^ People appreciate how staff work with them, they are free of charge and offer more than just needle exchange. But there are important barriers. Opening hours are an issue for some, though these vary across services/regions. Used equipment must usually be returned to receive sterile equipment (ibid 6§). There is some discretion here, as it is recognised that there are situations when a needle might not be returned, and NSP providers have relied more and more on this discretion.^3^ Critically, Swedish NSP is not anonymous. All clients must be registered in the relevant region where the NSP is located (ibid 6§). In other countries, anonymity is seen as a cornerstone of low-threshold NSP delivery. From this perspective, perhaps NSP in Sweden is already not low threshold, meaning the priority given to this principle was unwarranted in this case. However, while not anonymous, NSP is *confidential.* And it is this criterion that most speaks to the specific concern raised by the authorities regarding the duty to report.

It is fair to say that NSP is low threshold, based on the criteria set out by Mofizul Islam et al., even if not as low as models in other countries. Low thresholds are not static or uniform. Thus, a key question is why must the same level of threshold apply to all clients, if we already see variation in what is accepted as ‘low threshold’ across services or countries? What would prevent dedicated protocols for under 18s being developed in Sweden, to include child protection duties?

## The best interests of the child and NSP

The best interests of the child are seen as a ‘general principle’ of the CRC, alongside *inter alia*, the child’s right to be heard (Articles 3 and 12, respectively). Both are closely bound up with issues of maturity. According to the UN Committee on the Rights of the Child, the best interests of the child are a tripartite principle. First, it is a substantive right in itself, meaning that the child has an actionable right to have their best interests taken into account in whatever process is in question. Second, it is an interpretive legal principle. If a legal provision has more than one possible interpretation (as is very often the case), the one that best serves the best interests of the child should be chosen. Finally, it is a rule of procedure, in that legal or policy processes must assess the possible positive or negative effects on various options. States must ‘explain how the right has been respected in the decision, that is, what has been considered to be in the child’s best interests; what criteria it is based on; and how the child’s interests have been weighed against other considerations, be they broad issues of policy or individual cases’ [46], para 6).

The principle did not arise explicitly in the preparatory works, so it is unclear how the best interests of the child was properly taken into consideration in the decision to retain the age restriction. However, the authorities were apparently balancing competing values, in this case the interests of those under 18 who might not have access to NSP, and protecting the low threshold service. According to the CRC, after all, the best interest of the child is ‘a’ primary consideration, not ‘the’ primary consideration. As Alston notes, ‘the drafters’ preference for the indefinite rather than the definite article…is intended to indicate that the child's best interests are not to be considered as the single overriding factor’ ([1], p. 12).

The balancing of low thresholds versus the interests of legal minors raises long-standing discussions in the literature about exactly what weight is to be given to the best interests of the child in any given situation. John Eekelaar has offered a helpful framework [12]. For Eekelaar, ‘a distinction should be drawn between decisions that are directly about children and decisions that affect children indirectly’ (ibid pp 99 & 100). In the former case, the focus is on ‘seeking the *best outcome for the child’* (emphasis in original). This involves a subjective assessment and ‘choosing what is best for *this* child in *these* circumstances’ (emphasis in original). For example, such cases may involve custody or a child’s medical care. In the latter, the challenge is to reach the best solution in a case or on a policy issue. ‘The child’s interests are relevant and are *a* primary consideration, but there may be others’ (ibid). An example could be sentencing a primary caregiver for a serious offence. The best interests of the child are here weighed against a societal interest in punishing the crime. Eekelaar recognises that this is a very broad distinction with possible grey areas, but it is nonetheless an important one, as in any given situation ‘[D]ecision-makers need to choose which characterization best reflects the process as the way the child’s interests are set against other interests will depend upon which characterization is adopted' (ibid pp 109 & 110).

Let us look again at the rationale for the age restriction. While the best interests principle did not explicitly arise, the interests of clients were weighed. First, injecting drug use among under 18s was recognised as a potential issue, and that the small population size should not itself affect the decision [36]. It was accepted that these individuals may need access, but that allowing such access would affect the low threshold of the service. Thus, a decision ‘affecting’ children was made—one in which the best interests of the child is one among a range of important factors to consider, but not determinative as to the best course of action *for that problem*.

The age restriction on NSP in Sweden illustrates that a tension between these two characterisations of best interests can arise. Here, a decision ‘affecting’ children has been taken in which protecting another value was seen to take priority. However, the necessary effect of this is that assessments for individuals, closer to the door of the service, cannot be undertaken. Thus, a decision ‘about’ children (the stronger form) is not possible due to a decision ‘affecting’ them (the weaker form). How, then, might the situation look if it were instead characterised as a decision ‘about’ children? The next section explores best interests assessments in individual cases as a more flexible threshold for access than an age restriction, while remaining true to child protection obligations.

## From age restriction to best interests assessments?

Best interests assessments are already applied in various policy settings, from medical care, to custody hearings, to unaccompanied refugee children. It is primarily a subjective process and depends on the situation, the prevailing evidence, as well as the child’s age and maturity ([35], pp. 62 & 63). It can also change over time with new evidence and as social norms develop ([32], p. 100). Critically, the child’s own views must be given due weight. According to the preparatory works for the Swedish Patient Act, the question of maturity should be determined *in each case* by healthcare staff. The subjectivity of such decisions meant that a clear age limit for consent would be inappropriate ([35], pp. 66 & 67). The child, as an active subject, must be able to express what they think is in their best interests ([42], p. 11). Thus, what is in the best interests of the child must be determined in each individual case ([35], p. 63; [52], p. 212), and what is in the child’s best interests is therefore not defined in legislation. In principle, then, such assessments offer more flexibility than the age restriction (which is an a priori rule for all), while remaining grounded in legal child rights standards.

There is no single ‘recipe’ for such assessments, but a number of steps and criteria have been developed. The UN Committee on the Rights of the Child has summarised certain ‘elements’ that must be taken into account in any best interests assessment [46], and which can flesh out what these steps might have to consider. It is helpful to relate some of these to the topic at hand.

*The child’s views*: The right to be heard (Article 12) is a central tenet of the child rights framework, foregrounding children as active agents. As the Committee notes ‘Any decision that does not take into account the child’s views or does not give their views due weight according to their age and maturity, does not respect the possibility for the child or children to influence the determination of their best interests’ ([46], para 53). Critically, ‘The fact that the child is very young or in a vulnerable situation …does not deprive him or her of the right to express his or her views, nor reduces the weight given to the child’s views in determining his or her best interests’ (ibid, para 54). A best interests assessment, then, involves a dialogue that respects and advances the agency of the child, which is not possible when an age restriction precludes service access.

*Care, protection and safety of the child:* Here, the Committee emphasises the holistic approach adopted within the CRC, focusing on the child’s overall well-being, not only protection from harm (ibid, para 71). Here, we see the need for different perspectives (social, psychological, medical), safe referrals and comprehensive care.

*The child’s right to health*: The Committee on the Rights of the Child states that ‘the advantages of all possible treatments must be weighed against all possible risks and side effects, and the views of the child must also be given due weight based on his or her age and maturity. In this respect, children should be provided with adequate and appropriate information in order to understand the situation and all the relevant aspects in relation to their interests, and be allowed, when possible, to give their consent in an informed manner’ (ibid, para 77). To some extent, this reasoning was considered, albeit implicitly, in the development of the NSP Act. In both 2006 and 2016, it was put forward that other forms of intervention were more appropriate. The difference is that a best interests assessment would do this *on an individual basis*.

Such assessments are already applied to NSP in other settings. The well-known English case of *Gillick* involved access to sexual and reproductive health advice, and condoms, without parental consent (Gillick v West Norfolk and Wisbech AHA, [18]). The Court set out a basic test for service providers to go through and which can be done fairly rapidly (often referred to as the ‘Fraser Guidelines’ after the relevant judge). Does the young person understand what is being provided or suggested, and the rationale for the products/service? Does the young person refuse to provide parental consent? Is the young person likely to continue risky behaviour or to remain in a risky environment? Is the young person’s physical or mental health likely to suffer if the products/ services are not provided? Given the above, are the young person’s best interests served by providing the products/services? This type of assessment for anyone under 16 is now part of guidance for NSP from the National Institute for Health and Care Excellence [53] in the UK (those over 16 are presumed to be capable of consenting). It could be the case that the NSP is the first contact regarding a legal minor’s drug use (though in the Swedish context this is unlikely). Even if this is not the case, a rapid assessment could be conducted as to whether provision of injecting equipment is in their best interests. Here, the right to health, the child’s views, and a version of the Fraser Guidelines can serve as a framework.

The duty to report cases of children at risk remains a legal requirement. But this, too, retains a degree of discretion. A best interests assessment may play a role in the decision as to whether the young person should be reported to social services. For example, the default situation could be that encountering a legal minor who inject drugs should trigger the duty to report. And a protocol could be developed by Social Services and NSP providers which accounts for scenarios in which this might not be the best course of action for the young person. For example, if this would at the time damage an early, tenuous therapeutic relationship. It must be borne in mind, moreover, that many, if not most, legal minors who inject will already be known to Social Services. This was acknowledged in the debates regarding the NSP Act in 2016 ([36], p. 31). It might be the case, however, that Social Services are in contact with the young person but do not know about the injecting behaviour. Thus, the duty to report might apply. But if someone is already known to be injecting, then from a harm reduction perspective seeking assistance is a positive step in reducing risk.

It is important to recall, moreover, that best interests assessments already apply once a minor is reported to Social Services. Responses to such reports may involve compulsory care, but it is already the rule that other interventions should be exhausted first, which could include ongoing access to the NSP as part of a comprehensive care plan. In addition, according to the relevant law, in any decision on compulsory care, the child’s best interests are ‘decisive’. At this level, the stronger form of the best interests test is already in place.

Characterising the situation as a decision ‘about’ children, in which the best interests of the individual child (legal minor) are determinative, sets up the possibility for replacing an age restriction with subjective tests. This, to be sure, presents a higher threshold for access than those over the age of majority, but does not necessarily damage the low threshold nature of the service over all. Indeed, there seems to be an important gap in the reasoning of the authorities in this regard. For all clients, except those under 18, the threshold is the same whether under 18s are permitted to access or not. Removing the age restriction lowers the registration threshold considerably. Thus, overall, the service becomes lower threshold than it currently is, when all possible clients are considered. Accepting that the same threshold level of services would remain unchanged for all clients bar those under 18, the development of dedicated protocols for under 18s should not damage this valuable service modality over all. It would instead adapt it to the legal, ethical and clinical reality of the situation of legal minors, paying due regard to competence, efficiency and trust.

## Challenges with best interests assessments

Replacing an age restriction with best interests assessments offers the possibility to resolve the tension between access for those under 18 and low threshold service provision, while adhering to child protection obligations. However, it is not without challenges. Any such assessments have to be done by someone based on some form of guidance. This section discusses three interrelated issues: indeterminacy of the principle; assessing competence; and staff perspectives and capacity.

A benefit of age restrictions is clarity. They are used in many areas of law, including consent to sex, for joining the military, and for voting. They are clear, place everyone on the same understanding of what is permissible, and can provide a form of legal protection. For some, including those working in harm reduction, ‘you have to draw the line somewhere’ (as arose in [48], p. 373). To replace an age restriction on access to needle exchange with best interests assessments is to introduce a degree of indeterminacy into what was previously a clear-cut situation. Even if the choices are clear to those making an assessment, there can be reasonable disagreement ‘about which choice is the best’ ([3], p. 7). For some, the open-endedness of the best interests principle can lead to any number of subjective factors being considered, leading to arbitrariness [6]. This indeterminacy is central to criticisms of the principle as being too vague to function properly as a legal standard (see [29]). On the other hand, however, indeterminacy is arguably what is necessary in complex individual situations for clinical and ethical decision-making. Given our inability to predict all outcomes, the certainty of the age restriction can itself become so restrictive as to also be a source of arbitrariness in specific cases.

A best interests assessment may have differing temporal perspectives. Is the assessment for the short or long term? ([29], p. 260). What may seem to be in a young person’s best interests today may cause harm later on. But how could one reliably predict longer-term outcomes, and how far into the future should one be required to look? Arguably a best interests assessment ‘at the door’ of an NSP is short term, and Fraser Guidelines above lean towards a short-term frame of reference. There need not be a conflict between short- and long-term goals, of course, but here we see the challenge of viewing situations holistically, and ‘focusing on the child’s overall well-being, not only protection from harm’ ([46], para 71). Indeed, a short-term avoidance of harm can lead to longer-term relationship building in health and social care.

A further challenge is assessing competence in practice, which is closely tied to the weight to be given to the minor’s views as to their own best interests. Maturity and related competence in healthcare settings depend upon the child’s ability to understand a) the information being provided and b) the consequences of their decision ([35], pp. 66 & 67). As the judgment in *Gillick* read, “The child must be capable of making a reasonable assessment of the advantages and disadvantages of the treatment proposed, so the consent, if given, can be properly and fairly described as true consent” [18]. A first challenge is how this should be determined for each individual, especially in a context when such capacity for those over a certain age is presumed. In seeking to protect minors in a given (controversial) context, a standard could be put in place where more is required from them than would be expected of an adult in similar circumstances. Archard and Skivenes suggest that a way to address this is to ask *why competence is in doubt*, and if this would also be a reason to doubt it for an adult ([3], pp. 10 & 11). For adults attending NSP, drug use is obviously not a reason to doubt competence. They are deemed capable of understanding the need for the service and the consequences of their decision, given their experiences with injecting. The question is if this should be different for someone under the age of legal majority.^4^

Each of the above challenges relate to staff perspectives and capacities, i.e. *who* is conducting the assessment. Best interests assessments are not merely empirical, scientific endeavours from which normative aspects can be removed. The views of those making assessments as to the appropriateness of NSP as an intervention for under 18s must be considered. Such views have long been voiced in NSP debates and will be both professionally and socially formed. In research from Canada, a great deal of ethical hesitation about allowing under 18s to access supervised injection facilities was evident among stakeholders, including people who use drugs, service providers and representatives of relevant authorities [48]. In addition to their views and perspectives, there is the issue of staff competence. Would staff feel that they have the relevant training to undertake a best interests assessment for NSP access given the complexities that could be involved? Indeed, a recent evaluation of Sweden’s compliance with the CRC indicated that staff in various areas of social policy did not feel comfortable interpreting child rights obligations ([39]:63, p. 80). Careful attention would be needed to address the views, concerns and experiences of service providers, including the professional and social contexts in which they work. There are existing tools that may be used for working through these issues (for example [19].

## Conclusion

This article has looked at the legal framework for NSP and child protection in Sweden, and the rationale for the current age restriction of 18 on access to NSP. While that rationale is to protect the low threshold status of the NSP, it has suggested that differential thresholds accounting for ethical, legal and clinical issues relating to under 18s are appropriate. The article has proposed replacing the current age restriction with more flexible best interests assessments. This can relieve the tension between child protection and low thresholds that justifies the current age restriction, using a standard already firmly embedded in Swedish law and practice. It has also addressed some important ethical and practical challenges with such a change that would need careful attention in any reform process. This work will help to inform later focus group interviews with service providers in Sweden, aiming to explore their views, concerns and proposals regarding appropriate interventions and support for legal minors who inject drugs.

## Data Availability

Not applicable. All documents are publicly available.

## Footnotes

1 ‘Injecting drug use among legal minors and the best interests of the child: Lived experiences, support and policy frameworks in high income countries’, Forte grant no. 2020-00451. Focusing on Sweden, Switzerland and Wales, the project involves analysis of data collection systems, comparative legal and policy analysis, interviews with young people who inject drugs, and focus group interviews with harm reduction and drug treatment service providers.

2 Personal communication with staff from Västra Götalandsregionen (VGR) based on a survey conducted with clients.

3 This has proven to be important during Covid, when there was good reason to prevent unnecessary visits to hospitals and health centres where NSP is located.

4 The requisite competence for access to NSP could be presumed for those over the age of 15, in line with other areas of Swedish law (noted above), and similar to the UK where such capacity is resumed for those over 16. However, given the relevant child protection legislation, this paper assumes some form of best interests assessment for anyone under 18.
