# Correction: Best interests and low thresholds: legal and ethical issues relating to needle and syringe services for under 18s in Sweden

**DOI:** 10.1186/s12954-023-00735-8

**Published:** 2023-01-17

**Authors:** Damon Barrett, Frida Petersson, Russell Turner

**Affiliations:** 1 School of Public Health and Community Medicine, Sahlgrenska Academy, University of Gothenburg, Gothenburg, Sweden; 2 Department of Social Work, University of Gothenburg, Gothenburg, Sweden

**Correction:** **Harm Reduction Journal (2022) 19:15** **https://doi.org/10.1186/s12954-022-00597-6**

Following publication of the original article [1], in this article the paragraph under the section “Needle and syringe programmes, and needle purchase” should read as follows:

Needles may be sold in pharmacies in Sweden for medical use. If the person is under 20, the purchaser must prove it is for their own or a family member’s health condition ([24], 4§). A breach of this provision may result in a fine for the pharmacy involved (ibid 9§). This additional requirement for sales was seen as appropriate against the background of the same lower age limit being applied at the time to needle exchange ([34], p. 23). However, as this has now been reduced to 18 (discussed below), the two pieces of legislation are out of sync ([36], p. 36).

The original article has been corrected.
